# 40 years of CEPARM: transforming amyloidosis related to transthyretin from neglect to recognition

**DOI:** 10.1055/s-0045-1809401

**Published:** 2025-06-20

**Authors:** Marcia Waddington Cruz, Marleide da Mota Gomes

**Affiliations:** 1 Universidade Federal do Rio de Janeiro, Faculdade de Medicina, Programa de Pós-Graduação em Clínica Médica, Rio de Janeiro RJ, Brazil.; 2 Universidade Federal do Rio de Janeiro, Instituto de Neurologia, Rio de Janeiro RJ, Brazil.; 3 Universidade Federal do Rio de Janeiro, Instituto de Psiquiatria, Laboratório de História da Psiquiatria, Neurologia e Saúde Mental, Rio de Janeiro RJ, Brazil.

**Keywords:** Amyloid Neuropathies, Familial, Prealbumin, Polyneuropathies, Cardiomyopathies, Liver Transplantation, Drug Therapy

## Abstract

Variant transthyretin amyloidosis with polyneuropathy (ATTRv-PN) and cardiomyopathy (ATTRv-CM), formerly known as familial *amyloidotic polyneuropathy* (FAP), is a severe, progressive disorder caused by mutations in the *transthyretin* ( *TTR* ) gene. Historically, FAP was considered a neglected disease due to its rarity and the limited understanding of its pathophysiology, which led to minimal research funding and few therapeutic options. The present article explores the transformative role of Centro de Paramiloidose Antônio Rodrigues de Mello (CEPARM), established in 1984, in elevating the status of FAP through significant advancements in research and treatment. Although CEPARM was not the sole catalyst for this shift, its contributions in liver transplantation, the development of pharmacological therapies, and holistic patient care have substantially improved the recognition and management of FAP. The article also examines CEPARM's impact on patient care, the ongoing challenges, and ethical considerations within the field.

## INTRODUCTION

Familial amyloidotic polyneuropathy (FAP), now more commonly known as *variant transthyretin amyloidosis* (ATTRv), encompassing polyneuropathy (ATTRv-PN) and cardiomyopathy (ATTRv-CM), is a progressive and life-threatening disorder caused by mutations in the *transthyretin* ( *TTR* ) gene. These mutations result in misfolded TTR proteins that form amyloid deposits, primarily affecting peripheral nerves in ATTRv-PN and the heart in ATTRv-CM. 1 2

Historically, FAP was regarded as a low-prestige disease, largely neglected due to its rarity and the limited understanding of its mechanisms. This lack of recognition led to minimal research funding and scarce treatment options. Hindhede and Larsen's work 4 highlights how disease prestige is shaped by technological and research advancements, reflecting the challenges FAP faced in gaining attention. However, growing interest from the pharmaceutical industry, coupled with an increased focus on rare diseases, has been pivotal in transforming its status and improving the outcomes. 3 4

Variant transthyretin amyloidosis was initially considered a disease confined to endemic foci in Portugal, Japan, and Sweden. However, the presence of late-onset cases in endemic and non-endemic areas has now been recognized, indicating that the disorder is more common than previously thought. 5 6

## THE EARLY YEARS OF CEPARM

**Figure 1:**
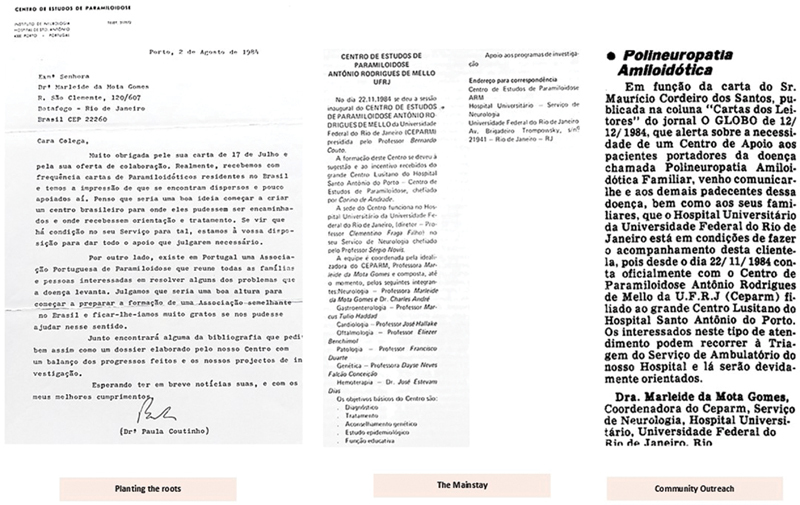
Founding Centro de Paramiloidose Antônio Rodrigues de Mello (CEPARM): initial meetings with Professor Paula Coutinho, center establishment, and community engagement featured in newspaper *O Globo* , December 1st, 1985. 7 8

Centro de Paramiloidose Antônio Rodrigues de Mello (CEPARM) was established in 1984 at Hospital Universitário Clementino Fraga Filho (HUCFF), Universidade Federal do Rio de Janeiro (UFRJ), by Marleide da Mota Gomes. The center was named in honor of Antônio Rodrigues de Mello, who first coined the term *familial amyloidotic polyneuropathy* . The establishment of the multidisciplinary CEPARM was driven by the need to offer specialized support and care for FAP patients, especially those of Portuguese descent, who were disproportionately impacted by this poorly-understood disorder ( Figure 1 ).

The establishment of CEPARM honored and extended the legacy of Corino Andrade, who first identified FAP, and Paula Coutinho, who expanded research into genetic neurological diseases. This center continued their pioneering work while introducing a multidisciplinary approach to meet the evolving needs of FAP patients. 7 8 9

Also in its foundational period, CEPARM encountered numerous challenges: FAP was largely unknown within the broader medical community, leading to a lack of awareness and resources. The center adopted a multidisciplinary approach, integrating various specialties to offer comprehensive care. Initial treatment strategies included plasmapheresis to remove abnormal prealbumin and the use of dimethyl sulfoxide (DMSO) to reduce amyloid deposits. These efforts were complemented by the development of care protocols, which guided specialists and promoted the center's services to patients. Despite these initiatives, CEPARM faced limitations in treatment efficacy and slow progress in understanding the disease. 6 7

**Figure 2:**
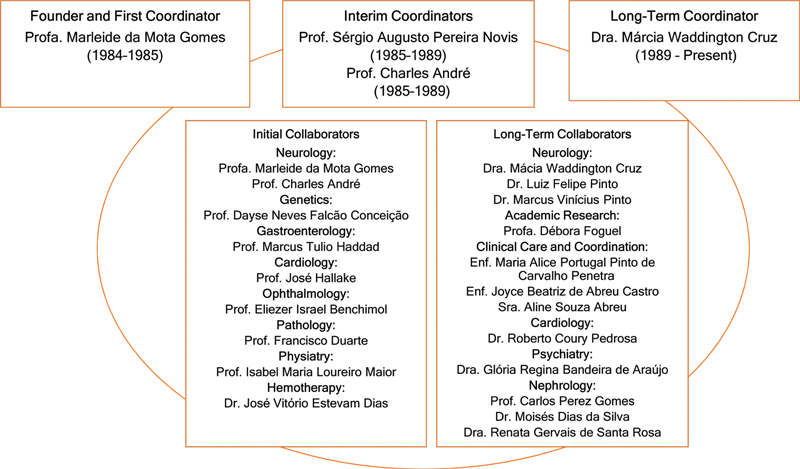
Evolution and featured collaboration of CEPARM.

The founder left this legacy to the institution and patients, pursued a master's degree at McMaster University, and subsequently spent 4 years at the Brazilian Ministry of Health, in Brasília. Other coordinators succeeded her, leading to the long administration by Márcia Waddington Cruz, a specialist in neuropathies, for 35 years. The interim administration involved the collaboration of Charles André and Sérgio Novis ( Figure 2 ). 10 11

## KEY MILESTONES IN THE EVOLUTION OF FAP TREATMENT

A significant breakthrough for CEPARM and FAP treatment occurred with the identification of *TTR* gene mutations as the cause of the disease. This discovery led to liver transplantation becoming a viable treatment option.

By replacing the main source of mutant *TTR* production, liver transplantation offered patients extended survival and improved quality of life. This advancement positioned CEPARM as a leading institution in FAP treatment, drawing global attention. 9

The development of pharmacological treatments further enhanced CEPARM's role in FAP research. Drugs such as the TTR stabilizer tafamidis, and TTR silencers such as patisiran, inotersen, vutrisiran, and eplontersen, were introduced, providing new therapeutic options that could stabilize or halt disease progression. These advancements were made possible through close collaboration with pharmaceutical companies, which recognized CEPARM as a key site for clinical trials. The center's involvement in these trials not only increased its prestige but also contributed significantly to the global understanding of FAP, making it a highly attractive area for research. 12 13 14 15 16 17 18 19 20

Table 1 summarizes the main CEPARM milestones.

**Table 1:** Milestones in CEPARM's Journey: advancements in ATTR research and treatment 10 11 12 13 14 15 16 17 18 19 20

Year	Event
1984	Founding of CEPARM: Established by Marleide da Mota Gomes at HUCFF/UFRJ to address the needs of patients with FAP, particularly those of Portuguese descent. Pioneered a multidisciplinary approach to care.
1989	Founding of ABPAR: Created to support amyloidosis patients and their families, strengthening community support for FAP treatment. CEPARM has been collaborating with ABPAR since its inception.
1993	The inaugural liver transplant in a PAF patient, in São Paulo, was performed by Professor Silvano Raia from USP on a patient of Professor Osvaldo Nascimento.
1997	First liver transplant in Rio de Janeiro by Professor Joaquim Ribeiro (UFRJ): Extended the availability of liver transplantation in Brazil, solidifying its role in FAP management.
2013	IX ISFAP: Hosted by CEPARM, it brought together global experts to discuss advancements in FAP research and treatment, marking the first and only ISFAP hosted in Latin America
2015	CEPARM facility established: A specialized facility for ATTR treatment and research was established at HUCFF-UFRJ, enhancing care and participation in global clinical trials.
2016	–Tafamidis approved by ANVISA for peripheral neuropathy: First pharmacological treatment approved in Brazil for FAP patients, marking significant progress in non-surgical management. CEPARM was the only Brazilian center involved in the international trial.– CEPARM's coordinator, Márcia Waddington, President of Scientific Committee of THAOS (2016–2019): Recognized globally with her appointment to the scientific committee of THAOS, a pivotal database to improve FAP treatment.
2017–2020	Tafamidis trial and approval for cardiomyopathy: Approved for treating cardiomyopathy associated with ATTR, offering new hope for cardiac patients. CEPARM was the first cardiac center elected for the ATTR-CM trial.
2018	Tafamidis officially included in the Brazilian Clinical Protocol and Guidelines for FAP treatment, ensuring broader patient access through public health services, in collaboration with CONITEC (Ministry of Health/Brazil).
2015–2024	–TTR silencers approved by ANVISA: Introduction of RNA-based therapies (e.g., patisiran, inotersen) aimed at reducing abnormal TTR protein production, revolutionizing FAP treatment. 2019: Clinical trials for next-generation silencers (e.g., vutrisiran, eplontersen) begin, focusing on more effective treatments with fewer side effects. CEPARM is actively involved as the coordinator center for these trials in Brazil.
	First- and second-generation TTR silencers trials: CEPARM participated in trials for both generations of TTR silencers, serving as the coordinator center, top patient recruiter, and consulted for CONITEC in establishing these therapies.
2008–2023	THAOS database contributions: Significant contributions made to the THAOS database, enrolling over 6 thousand patients worldwide and carriers, shaping the understanding of ATTR and its treatment.
2024	By 2024, more than 800 patients had gained access to tafamidis, underscoring CEPARM's impact on providing life-extending treatments. CEPARM provides multidisciplinary care for over 450 patients from various parts of Brazil and Latin America.

Abbreviations: ANVISA, Agência Nacional de Vigilância Sanitária; APBAR, Associação Brasileira de Paramiloidose; ATTR, transthyretin amyloidosis; ATTRv-CM, Variant transthyretin amyloidosis with cardiomyopathy; CEPARM, Centro de Paramiloidose Antônio Rodrigues de Mello; CONITEC, Comissão Nacional de Incorporação de Tecnologias; FAP, familial amyloidotic polyneuropathy; HUCFF, Hospital Universitário Clementino Fraga Filho; ISFAP, International Symposium on FAP; THAOS, Transthyretin Amyloidosis Outcomes Survey; TTR, transthyretin; UFRJ, Universidade Federal do Rio de Janeiro; USP, Universidade de São Paulo.

## CURRENT STATUS OF CEPARM

Today, CEPARM and other centers in Brazil are internationally recognized for their contributions to FAP research and treatment. Applying innovative protocols and multidisciplinary approach have set new standards for patient care, transforming FAP from a neglected condition into a focal point of high research interest. The success of CEPARM is a testament to its commitment to advancing scientific knowledge and improving patient outcomes. The center's work has extended the lives of FAP patients and provided comprehensive care that addresses the physical, psychological, and social aspects of the disease.

## IMPACT ON RESEARCH AND PATIENT CARE

The efforts made by CEPARM have significantly enriched the broader field of amyloidosis research, particularly in understanding and treating ATTR. The center's involvement in clinical trials has provided valuable data that has been instrumental in developing new treatments. These advancements have not only benefited FAP patients, who only have access to new and highly-expensive drugs being enrolled in clinical trials but also deepened scientific insights into ATTR.

## CHALLENGES AND ETHICAL CONSIDERATIONS

Centro de Paramiloidose Antônio Rodrigues de Mello faces the ongoing challenge of balancing innovation and ethical responsibility, especially as its pharmaceutical partnerships continue to expand. Although these collaborations are essential to advance new treatments, they can also introduce potential conflicts of interest. To safeguard CEPARM's reputation, maintaining an unwavering commitment to patient care and upholding strict ethical standards is vital.

With the original long-serving leadership, succession planning and staff development have become crucial to preserve the center's legacy of excellence in both care and research. Preparing future leaders to carry forward CEPARM's values amid an evolving healthcare landscape is key to sustaining its mission.

The rising interest in rare diseases provides an opportunity for CEPARM to strengthen its position by partnering with multidisciplinary centers. This approach, similar to the Division of Rare Diseases Research Innovation (DRDRI) of the United States National Institutes of Health (NIH), 3 can enhance resources and collaboration, helping CEPARM address ethical challenges, ensure leadership continuity, and optimize patient care.

In conclusion, the transformation of FAP from a neglected disease to a prominent research focus has been influenced by several factors, with CEPARM playing a pivotal role in this evolution.
